# Solution Processed Hybrid Polymer: HgTe Quantum Dot Phototransistor with High Sensitivity and Fast Infrared Response up to 2400 nm at Room Temperature

**DOI:** 10.1002/advs.202000068

**Published:** 2020-05-10

**Authors:** Yifan Dong, Mengyu Chen, Wai Kin Yiu, Qiang Zhu, Guodong Zhou, Stephen V. Kershaw, Ning Ke, Ching Ping Wong, Andrey L. Rogach, Ni Zhao

**Affiliations:** ^1^ Engineering Research Center of Nano‐Geomaterials of Ministry of Education Faculty of Materials Science and Chemistry China University of Geosciences Wuhan 430074 China; ^2^ Department of Electronic Engineering The Chinese University of Hong Kong Shatin, New Territories, 999077 Hong Kong SAR China; ^3^ Department of Materials Science and Engineering and Centre for Functional Photonics (CFP) City University of Hong Kong Kowloon Hong Kong SAR 999077 China; ^4^ School of Materials Science and Engineering Georgia Institute of Technology Atlanta GA 30332 USA

**Keywords:** HgTe quantum dots, infrared photodetection, IR photodetection, phototransistors, poly(3‐hexylthiophene)

## Abstract

Narrow bandgap semiconductor‐based photodetectors often suffer from high room‐temperature noise and are therefore operated at low temperatures. Here, a hybrid poly(3‐hexylthiophene) (P3HT): HgTe quantum dot (QD) phototransistor is reported, which exhibits high sensitivity and fast photodetection up to 2400 nm wavelength range at room temperature. The active layer of the phototransistor consists of HgTe QDs well dispersed in a P3HT matrix. Fourier‐transform infrared spectra confirm that chemical grafting between P3HT and HgTe QDs is realized after undergoing prolonged coblend stirring and a ligand exchange process. Thanks to the shifting of the charge transport into the P3HT and the partial passivation of the surface traps of HgTe QDs in the blend, the P3HT: HgTe QD hybrid phototransistor shows significantly improved gate‐voltage tuning, 15 times faster response, and ≈80% reduction in the noise level compared to a pristine HgTe QD control device. More than 10^11^ Jones specific detectivity (estimated from the noise spectral density measured at 1 kHz) is achieved at room temperature, and the response time (measured at 22 mW cm^−2^ illumination intensity) of the device is less than 1.5 µs. That is comparable to commercial epitaxially grown IR photodetectors operated in the same wavelength range.

## Graphical abstract

A hybrid poly(3‐hexylthiophene) (P3HT): HgTe quantum dot (QD) bulk‐heterojunction phototransistor is reported. By chemically grafting P3HT around the QDs, an active layer morphology with QD sensitizers dispersed in a continuous P3HT phase is obtained. The structure significantly reduces noise, leading to more than 10^11^ Jones room‐temperature specific detectivity in a wavelength range up to 2300 nm.

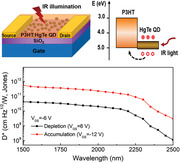

Infrared (IR) light photodetection has important and broad applications in telecommunications,^[^ ^1^ ^]^ biomedical imaging,^[^ ^2^ ^]^ night vision for military or civil surveillance,^[^ ^3^ ^]^ gas sensing,^[^ ^4^ ^]^ and chemical spectroscopic analysis.^[^ ^5^ ^]^ However, the mainstream photosensing materials used in commercial photodetection technologies still rely on expensive and wafer/die size‐limited epitaxial growth processes and have poor compatibility with silicon integrated circuit technologies (for sensing wavelengths beyond 1.1 µm).^[^ ^6^ ^]^ The colloidal HgTe quantum dots (QD) on the other hand, with tunable spectral response spanning the entire near‐to‐mid infrared range,^[^ ^7^ ^]^ intrinsically low Auger recombination rate,^[^ ^8^ ^]^ and an easy solution processability which allows for high‐throughput production and the use of flexible substrates, have the potential to revolutionize the next generation of IR photodetectors.^[^ ^1^, ^9^, ^10^, ^11^ ^]^

In the past few years, many research efforts on colloidal HgTe QD based photodetectors concerned both i) wavelength sensing range extension (i.e., from short‐wave infrared (SWIR, normally defined as 1−3 µm)^[^ ^4^, ^12^, ^13^, ^14^ ^]^ to mid‐wave infrared (MWIR, normally defined as 3−5 µm),^[^ ^15^, ^16^, ^17^ ^]^ or even long‐wave infrared (LWIR, normally defined as 8−12 µm))^[^ ^18^ ^]^ and ii) device structure optimization (i.e., from photoconductor^[^ ^12^, ^15^ ^]^ to phototransistor^[^ ^4^, ^13^ ^]^ to photodiode^[^ ^14^, ^16^, ^17^ ^]^). Room temperature operated high sensitivity (specific detectivity D* > 10^10^ Jones (cm Hz^1/2^ W^−1^ in international system of units (SI)) HgTe QD based photodetectors have been respectively demonstrated with phototransistors^[^ ^4^, ^13^ ^]^ and photodiodes^[^ ^17^, ^19^ ^]^ in the SWIR wavelength range. However, in most reported cases, the HgTe QD layers are functioning as both IR absorber and charge transport media. Due to the strong thermal excitation in narrow bandgap materials and the high surface defect density of QDs, the noise level of HgTe QD based IR photodetectors is intrinsically high, which greatly hinders the further improvement of the room temperature detectivity. This situation becomes even worse at longer sensing wavelengths. All of the reported HgTe QD based photodetectors in the MWIR wavelength range still required cryogenic temperatures to reach high sensitivities.^[^ ^15^, ^16^, ^17^, ^20^ ^]^ This obstacle is also met by most commercial products operating in the same wavelength range. The consequent and necessary extra cooling components not only increase the fabrication cost but also limit the scope for miniaturized applications in portable imagers, spectrometers, and so on.

To overcome this bottleneck, one of the possible choices is to hybridize the HgTe QDs with other material systems. In 2017, N. Huo et al. reported a HgTe QD/TiO_2_/MoS_2_ heterojunction phototransistor with HgTe QDs as the IR absorber and 2D MoS_2_ as the charge transport layer, achieving up to 10^12^ Jones room temperature detectivity at 2 µm.^[^ ^13^ ^]^ That can be attributed to the significant gate voltage controlled noise level with the assistance of a buffer layer of TiO_2_. However, considering the difficulties in large‐scale production and patterning of the 2D materials, other materials, which are compatible with solution‐phase and high‐throughput production techniques (e.g., inject printing, spray coating), are still highly desirable alternatives.

Embedding HgTe QDs in a conductive polymer matrix, forming an organic–inorganic hybrid photodetector, is a promising method to mitigate the above limitations.^[^ ^21^, ^22^, ^23^, ^24^, ^25^, ^26^ ^]^ Poly(3‐hexylthiophene) (P3HT), as a well‐established conjugated conductive polymer with great electronic properties and mechanical and chemical stabilities, has already been shown to offer marked improvements in the performance of other organic–inorganic hybrid photovoltaic and photodetector devices.^[^ ^21^, ^22^, ^23^, ^24^ ^]^ On the one hand, the bulk heterojunction structure between the P3HT and QDs provides a more versatile method to control the charge separation and transport;^[^ ^22^, ^23^ ^]^ on the other hand, the interaction between the surface defects of QDs and P3HT provides tunability in device performance optimization, especially in photodetection applications.^[^ ^24^ ^]^ However, obtaining a controllable bicontinuous percolation network (for each polarity of charge carrier) and a well‐defined interface between the QDs and the polymer matrix remains challenging. Especially, because of the surface energy of the QDs is different from that of the polymer, a phase separation between the P3HT and the nanocrystals was observed in previous publications,^[^ ^21^, ^25^ ^]^ and this may lead to poor morphology and charge extraction in the hybrid layer in practice. Therefore, it is of importance to manipulate the interface between the HgTe QDs and the P3HT to realize a hybrid layer with uniform distribution and a high loading density of HgTe QDs without aggregation.

Here, we demonstrate a P3HT: HgTe QD hybrid phototransistor with high sensitivity photodetection up to 2400 nm at room temperature. With a carefully controlled coblend stirring and ligand exchange process, the nanoscale morphology and the charge transport of the hybrid layer are optimized, which result in a uniform phase distribution. Fourier‐transform infrared spectroscopy (FTIR) spectra confirm that coordination bonds are formed between P3HT and the HgTe QDs indicating chemical grafting. Compared to the pristine HgTe QD control device, the P3HT: HgTe QD hybrid phototransistor shows significantly better gate‐voltage tuning, 15 times faster photoresponse, and close to one order of magnitude reduction in the noise level. That can be explained by a shifting of the charge transport path from QDs to the P3HT in the layer and the partial passivation of the surface traps of the QDs with P3HT. More than 10^11^ Jones specific detectivity and less than 1.5 µs response time are achieved at room temperature, which are among the highest recorded values to date for HgTe QD based SWIR photodetectors.

The fabrication strategy of the P3HT: HgTe QD hybrid layer is illustrated in **Figure** **1**a. HgTe QDs are synthesized by an aprotic solvent gas‐injection method^[^ ^27^ ^]^ and provide a material with a photoluminescence (PL) peak at around 2400 nm (Figure S1, Supporting Information). After several rounds of precipitation to wash out impurities/unreacted precursors, the dots are capped with 1‐dodecanethiol (DDT) ligands and stabilized in toluene. Subsequently, with gentle heating at 40 °C and prolonged coblend stirring, P3HT is slowly dissolved and become well‐mixed with the HgTe QD solution. A stirring time of 4−6 h was found to be optimal. A shorter stirring time may lead to ineffective coordination and a tendency toward phase separation between the P3HT and the QDs (discussed later). A longer time may reduce the PL quantum yield of the QDs due to long‐term heat exposure whilst in solution. The long alkyl chains of DDT make the HgTe QDs stable and easily dispersible in the P3HT/solvent mixture and at the ligand chain/polymer molecular level the two organic materials are very compatible. However, the conductivity of the hybrid film is still affected by the large spacing enforced by the long chain ligands between the QDs. To facilitate interparticle charge transport, the DDT is partially replaced by a shorter ligand, 1,2‐ethanedithiol (EDT), in a solid film phase ligand exchange treatment. At the end of the fabrication procedure, a gentle annealing is applied after the spin deposition and the ligand exchange process to fully remove the extra solvent and to help the hybrid's constituents relax via a self‐assembling and packing process.

**Figure 1:**
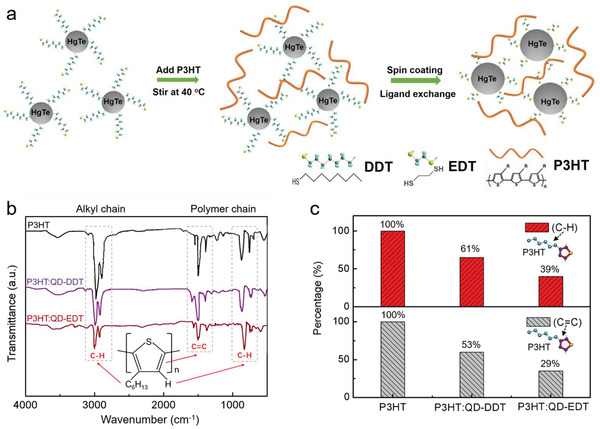
a) Schematics of the fabrication strategy of P3HT: HgTe QD hybrid films. b) Normalized FTIR spectra and the characteristic P3HT vibration peaks in pristine P3HT film, P3HT: HgTe QD hybrid film before (P3HT: QD‐DDT) and after ligand exchange (P3HT: QD‐EDT). c) Comparison of the corresponding absorption characteristic peaks (C=C peak at 1508 cm^−1^ and C—H peak at 2926 cm^−1^) in the above mentioned films.

To reveal the chemical nature of the coordination during the hybridization process described above, the FTIR spectra of the P3HT: HgTe QD hybrid layer before (as P3HT: QD‐DDT) and after the ligand exchange (as P3HT: QD‐EDT), with a comparison to the pristine P3HT film, were measured and are shown in Figure 1b. The concentrations of the P3HT are kept the same in both the pristine and co‐blend solutions. As previously reported,^[^ ^24^ ^]^ the strengths of the vibration peaks of P3HT will be greatly reduced if coordination bonds to the QDs are formed. Moreover, the reduction percentage is dependent on the distances between the corresponding bonds to the coordination site (in our case, a S—Hg bond is formed). Therefore, we normalized the FTIR spectra with the out‐of‐skeleton C—H vibration peak in the thiophene ring (821 cm^−1^),^[^ ^28^ ^]^ which is less affected by the formation of the S—Hg bond. As compared to the pristine P3HT film, the vibration peak intensities of the C=C bond (1508 cm^−1^) in the thiophene ring and the C—H bond (2926 cm^−1^) in the alkyl chain are both obviously reduced in hybrid films, no matter whether before or after the ligand exchange. The reduction percentages of the corresponding peaks are calculated and are shown in Figure 1c. Considering the absorption peak intensity of pristine P3HT as 100%, the C=C vibration of P3HT: QD‐DDT hybrids is reduced to about 53%, which is indicative of coordination bonds between the P3HT and QDs forming after the coblend stirring stage. The further intensity reduction (to 29%) of the same peak after ligand exchange (from DDT to EDT) indicates that the ligand exchange process evidently enhances the chemical grafting. The C—H vibration peak in the alkyl chain shows similar reduction trends, which also supports the conclusion that coordination bonds between the P3HT and QDs are formed during the coblend and ligand exchange process. Compared to the C=C bond (with a position immediately adjacent to the S—Hg bond), the relatively smaller peak intensity reduction of the C—H vibration in the alkyl chain matches well with its larger distance to the S—Hg bond site. It is worth mentioning that the peak intensity of the out‐of‐skeleton C—H vibration in the thiophene ring also reduces with the formation of S—Hg bond. That is, the peak intensity reductions shown in Figure 1b are underestimated using the out‐of‐skeleton C—H vibration as a constant normalization; in reality the chemical grafting induced changes can be even stronger than calculated here.

The cross‐sectional scanning electron microscope (SEM) image of the P3HT: HgTe QD hybrid layer deposited on a silicon wafer is provided in **Figure** **2**a, showing that HgTe QDs are well dispersed across the P3HT layer without phase separation. The corresponding energy‐dispersive X‐ray spectroscopy mappings of Si (Figure 2b), Hg, and Te elements (Figure 2c,d) confirm the uniform distribution of HgTe QDs in the P3HT matrix. The stirring time of the coblend solution is essential for the uniform distribution of HgTe QDs in the P3HT matrix. As shown in Figure S2 in the Supporting Information, clear phase separation between the HgTe QD and P3HT layers is observed in the hybrid film deposited after a much shorter 1 h stirring duration. By increasing the stirring time of the coblends, the phase separation tendency is reduced and the HgTe QDs are well dispersed into the P3HT layer after 4 h stirring.

**Figure 2:**
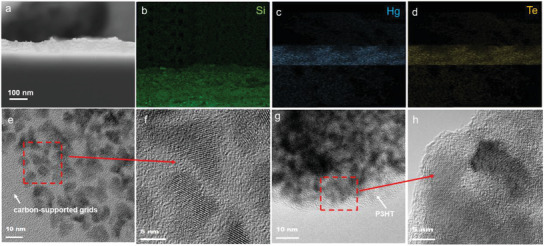
a) SEM image of the cross‐section of P3HT: HgTe QD hybrid phototransistor. b‒d) Corresponding energy‐dispersive X‐ray spectroscopy (EDX) profiles measured from the cross‐section of P3HT: HgTe QD hybrid phototransistor for the three detected elements: Si, Hg, and Te, respectively. e) TEM and f) HRTEM images of the HgTe QDs. g) TEM and h) HRTEM images of the P3HT: HgTe QD hybrids.

The transmission electron microscopy (TEM) images and the high resolution transmission electron microscopy (HRTEM) images of the pristine HgTe QDs and P3HT: HgTe QD hybrids are shown in Figure 2e–h. Figure 2e is the TEM image of HgTe QDs, which have mostly triangular shapes with sizes ranging from 5 to 6 nm. The HRTEM image in Figure 2f shows obvious lattice fringes of the HgTe QDs, indicating their high crystallinity. The TEM and HRTEM images of P3HT: HgTe QD hybrids are respectively shown in Figure 2g,h. The HgTe QDs, which are uniformly dispersed in the P3HT polymer matrix, still maintain their crystalline quality, which is also confirmed by the X‐ray diffraction patterns (Figure S4, Supporting Information).

After the uniformity of the films and the crystallinity of QDs were confirmed, a phototransistor was fabricated by direct deposition of the P3HT: HgTe QD hybrid layer on a standard heavily doped silicon substrate (as a gate contact) with a thin insulating silicon dioxide surface layer (as dielectric), as shown in **Figure** **3**a. The gold interdigital electrodes were photolithographically prepatterned before the P3HT: HgTe QD layer deposition, working as source and drain contacts. A multiple spin deposition and ligand exchange fabrication strategy was adopted to produce a thick film for better conductivity and light absorbance (see Figure S5, Supporting Information, for thickness and morphology measurement). We also fabricated pristine P3HT and HgTe QD transistors in the same way and with the same type of substrates for working mechanism comparisons. The typical transfer characteristics of the pristine P3HT, HgTe QD, and P3HT: HgTe QD hybrid transistors under dark conditions are shown in Figure 3c. The transfer and gate leakage current comparisons of the corresponding devices are shown in Figure S7 in the Supporting Information.

**Figure 3:**
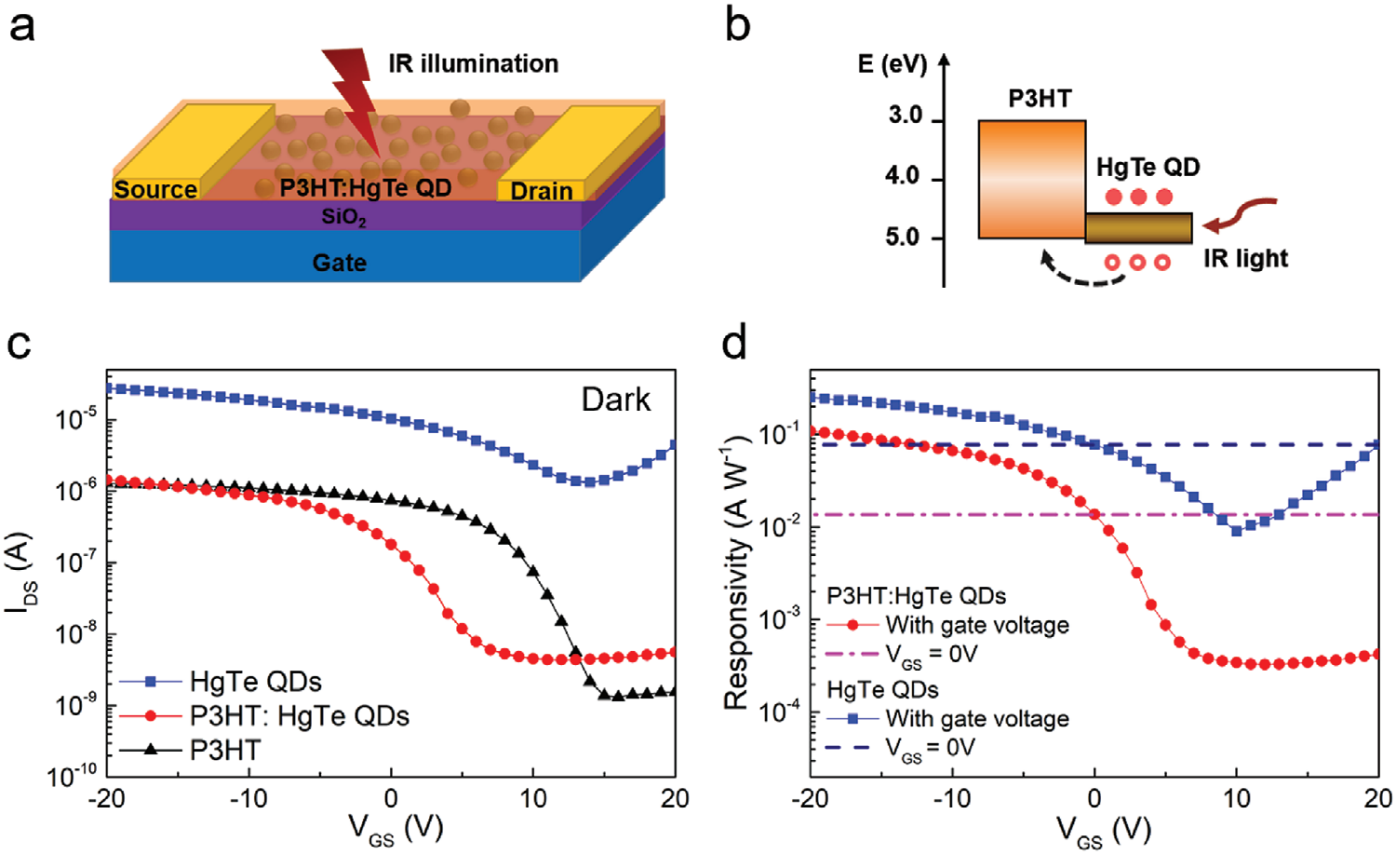
Schematics of a) device structure of the P3HT: HgTe QD hybrid phototransistor and b) band alignment and charge transfer between P3HT and HgTe QDs. c) Typical transfer characteristics of pristine P3HT, HgTe QD, and P3HT: HgTe QD hybrid phototransistors under dark conditions. The curves are measured with *V*_DS_ = −5 V in a forward scan, with *V*_GS_ scanned from +20 to −20 V. d) Gate‐voltage‐dependent responsivity of the HgTe QD based phototransistor and P3HT: HgTe QD hybrid phototransistor. The responsivity at *V*_GS_ = 0 V is depicted for comparison. Illumination level: 1550 nm, 22 mW cm^−2^. All data were obtained at room temperature (298 K).

The pristine HgTe QD transistor that we fabricated showed high conductivity and an ambipolar property, with a dominant p‐type mobility, *μ*_h_ ≈ 5 × 10^−3^ cm^2^ V^−1^ s^−1^, and an on/off ratio ≈20. By shifting the charge transport path into P3HT, the electron transport of the HgTe QDs is depressed with a prompt marked improvement in the gate‐voltage tuning (on/off ratio ≈330). However, considering the low annealing temperature adopted in the device fabrication, the P3HT in the pristine and hybrid films is amorphous, as the TEM image in Figure 2h indicates. The conductivity of the pristine P3HT film is much lower than the HgTe QD film with p‐type mobility *μ*_h_ ≈ 5 × 10^−4^ cm^2^ V^−1^ s^−1^. The transport property of the hybrid film is similar to the pristine P3HT film (also with p‐type mobility *μ*_h_ ≈ 5 × 10^−4^ cm^2^ V^−1^ s^−1^), which indicates that the charge transport path in the hybrids is dominated by the P3HT. With the energy diagram adapted from previous reports,^[^ ^24^, ^29^ ^]^ the band alignment between the P3HT and HgTe QDs is depicted in Figure 3b, which belongs to the Type II alignment. Under IR illumination, the photoexcited holes in HgTe QDs can be efficiently transferred into the P3HT matrix, leaving the electrons blocked by the energy barrier of P3HT. This mechanism should favor overall photocurrent improvement by facilitating the charge separation and extraction processes.

We respectively characterized the optical response of the pristine HgTe QD and hybrid phototransistors, with gate‐voltage dependent responsivities shown in Figure 3d. Biasing the gate at −20 V (accumulation mode), the responsivity of the hybrid devices is raised by ≈20 times compared to the *V*_GS_ = 0 V operation (photoconduction mode). This is much higher than for the pristine HgTe QD phototransistor (approximately three times) under the same conditions. However, the overall responsivity of the hybrid phototransistor is still lower than for the pristine QD based device. That can be analyzed in terms of the photoconductive gain mechanism. With the p‐type dominated charge transport property, the photoconductive gain of the hybrid phototransistor can be roughly evaluated with *G* = *τ*_c_/*τ*_t_, where *τ*_c_ is the average trapping time of the photogenerated electron and *τ*_t_ is the transit time of the photogenerated hole drifting between the source and drain electrodes under external bias. Compared to the pristine HgTe QD phototransistor, the lower hole mobility (indicating a longer transit time, *τ*_t_) and the reduced average charge trapping time *τ*_c_ (which will be discussed in the following) of the hybrid device both cause a loss in photoconductive gain.

The light intensity dependent responsivities of P3HT: HgTe QD and pristine HgTe QD phototransistors in the accumulation mode are characterized and compared in **Figure** **4**a. At as low as 2.2 µW cm^−2^ illumination, the pristine HgTe QD and P3HT: HgTe QD hybrid phototransistors reach remarkably high responsivities of 12.4 and 5.2 A W^−1^, respectively. However, the responsivities of both devices decrease with increasing light intensity because of the loss in photoconductive gain. Figure 4b shows the power law dependences of the photocurrent (*I*_ph_) with the light intensity (*I*), i.e., *I*_ph_ *∝ I^*α*^*. At high light intensities, both *α* values of the pristine QD and hybrid phototransistors are close to 0.5, corresponding to a bimolecular recombination dominated loss process.^[^ ^30^ ^]^ At low light intensity levels, in both cases *α* increased and approached to ≈1, indicating a transition from bimolecular recombination to trap‐assisted recombination when the photogenerated carrier density was reduced. Moreover, the transition from bimolecular to trap‐assisted recombination in P3HT: HgTe QD hybrid phototransistors occurred at one order of magnitude lower illumination level compared to the pristine HgTe QD device, suggesting there is a relatively lower trap density in the hybrid device.

**Figure 4:**
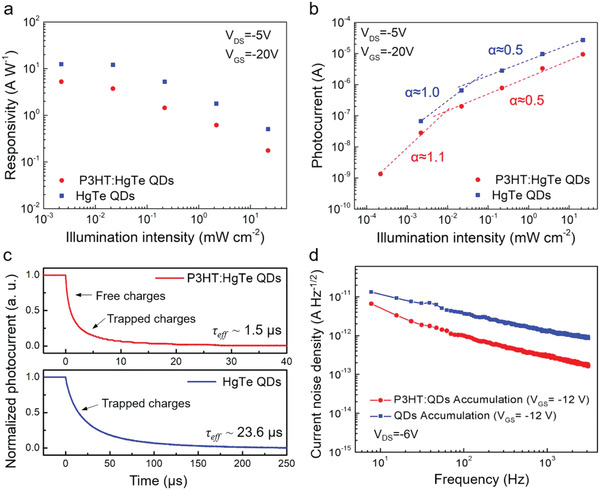
Comparisons of the typical pristine HgTe QD and P3HT: HgTe QD hybrid phototransistors for a) light intensity dependent responsivity, b) light intensity dependent photocurrent (shown as a log−log fit), c) photocurrent transient response, and d) current noise spectral density under accumulation operation. Samples in (a)–(c) were all illuminated with a 1550 nm laser. Illumination level in (c): 22 mW cm^−2^. All data were measured at room temperature (298 K).

We also characterized and compared the transient photocurrent response of both devices in Figure 4c. The hybrid phototransistor exhibited a fast response speed with an effective decay time *τ*_eff_ ≈1.5 µs (The measurement performed with a 1550 nm laser at 22 mW cm^−2^ illumination intensity.), which is 15 times faster than that of the pristine QD devices *τ*_eff_ ≈23.6 µs. The effective decay time constant is defined as the time needed to fall to the *e^−1^* (≈37%) of the original amplitude observed in the decay tail, which is also referred as decay time or recovery time for different photodetectors.^[^ ^31^, ^32^, ^33^, ^34^ ^]^ The photocurrent decay of the hybrid phototransistor is dominated by the fast charge extraction of free charges in the initial stage of the transient and by the slow detrapping process of trapped charges in the later stage (Figure S9, Supporting Information). However, under the same illumination level, the slow detrapping of trapped charges dominates the photocurrent decay of the pristine QD device whilst the fast component is not observed in this device. That indicates that the extraction of free charges is suppressed due to the large density and depth of the trap states in the pristine QD devices, which corresponds well with the light intensity dependent fitting observed. On the one hand, the bulk‐heterojunction of the hybrid device facilitates charge extraction by reducing the recombination loss within QDs. On the other hand, considering the formation of coordination bonds between P3HT and QDs in the hybrids indicated by FTIR spectra, the trap state density reduction in the hybrid device should be attributed to the partial passivation of the surface traps of the HgTe QDs by P3HT. It is noted that the reduced electron trap density also decreases the responsivity to some extent.

Although the responsivity of the pristine HgTe QD phototransistor is higher than the P3HT: HgTe QD hybrid one, the specific detectivity, both considering the responsivity and the noise level, is the most important figure‐of‐merit to compare the sensitivity of different photodetectors. As shown in Figure 4d, we respectively measured the current noise spectral densities of the pristine HgTe QD and P3HT: HgTe QD hybrid phototransistors under accumulation operation. The noise level in the hybrid device is about 20% of the one in the pristine QD sample. By shifting the predominant charge hopping medium from the QDs to the conducting polymer, the noise level is overall suppressed. The noise spectrum of both devices shows a dominated flicker noise (1/*f*) trend,^[^ ^35^ ^]^ which is commonly observed in other thin film transistors in the low frequency range.^[^ ^36^, ^37^ ^]^ In general the flicker noise can be caused by carrier density fluctuation related to trapping and detrapping processes or carrier mobility fluctuation related to random lattice scattering or other processes.^[^ ^36^, ^38^ ^]^ Presently, we cannot distinguish which mechanism dominates the flicker noise. Further studies involving temperature‐dependent and gate‐voltage dependent measurements may help to elucidate the noise mechanism. The surface passivation of the QD surface by P3HT is an efficient way to control the flicker noise, which was previously observed in other devices.^[^ ^24^ ^]^ We note that the shot noise level estimated from the *I*–*V* curve at Figure 3c is around, which is larger than the noise spectral density measured here. This deviation could be due to the different measurement modes and measurement setup (see the Experimental Section for details). In particular, the noise measurement was done after stabilizing the device at a constant voltage, while the *I*–*V* measurement was done by sweeping the voltage from positive to negative direction; the latter tends to give a higher apparent current.

With the measured noise level (Figure 4d, at 1 kHz) and responsivity (Figure 3d), the specific detectivities of pristine HgTe QD and P3HT: HgTe QD hybrid phototransistors can be respectively calculated with D* =* R* (Area)^1/2^/*I*_n_, where *R* is the responsivity, Area is the sensing area, and *I*_n_ is the current noise density. Under accumulation operation and 22 mW cm^−2^ illumination, the detectivity of the hybrid phototransistor is 1.2 × 10^10^ Jones, which is clearly higher than that of the pristine QD device at 7.6 × 10^9^ Jones. At lower light intensities, the detectivity will further increase due to the light intensity dependence of the responsivity (as shown in Figure 4a). This effect can be seen in **Figure** **5**, which will be discussed later. In general, our results suggest that by efficiently dispersing the HgTe QDs into the P3HT matrix, we can significantly reduce the noise level of the phototransistor. Hence, even the responsivity of the hybrid device is lower than the pure QD device, the detectivity of the former is increased by 50%.

**Figure 5:**
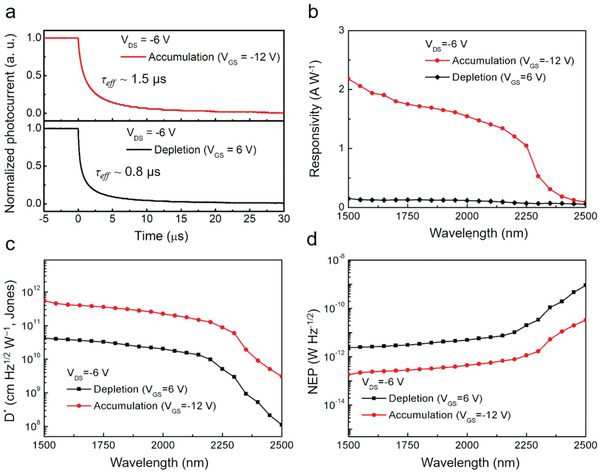
Optoelectronic characteristics of the P3HT: HgTe QD hybrid phototransistor: a) temporal response; and b) wavelength‐dependent responsivity; c) specific detectivity (at 1 kHz); and d) noise‐equivalent power (at 1 kHz) spectra, operating in the depletion mode (black) and accumulation mode (red), respectively. All data are measured at room temperature (298 K).

Finally, the room temperature photodetection performances of a P3HT: HgTe QD hybrid phototransistor were characterized, both in accumulation and depletion modes. As the temporal responses in Figure 5a show, the effective decay time of the phototransistor operated in accumulation and depletion mode is *τ*_eff_ ≈1.5 µs and *τ*_eff_ ≈0.8 µs, respectively. Accordingly, the 3 dB bandwidth *f*_3dB_ can be respectively evaluated in ≈100 and ≈200 kHz range (taking *f*_3dB_ = 1/(2*πτ*_eff_)).^[^ ^31^ ^]^ These results compare favorably with the fastest response speeds of the previously reported HgTe QD based photodetectors operated in the 2 µm plus wavelength range, which in those cases were provided for photodiode structures.^[^ ^16^, ^17^ ^]^ Figure 5b shows the wavelength‐dependent responsivity spectra of the phototransistor operated in both modes. Under accumulation operation the device exhibited more than 1.5 A W^−1^ responsivity just beyond 2000 nm. With the current noise spectral densities of the hybrid phototransistor measured in accumulation and depletion operation (Figure S10, Supporting Information, at 1 kHz), the wavelength‐dependent D* and noise‐equivalent power (NEP) spectra of the hybrid phototransistor are respectively calculated and shown in Figure 5c,d. More than 10^11^ Jones detectivity and lower than 10^−12^ W Hz^−1/2^ NEP were obtained in accumulation mode. The room temperature high sensitivity detection (taken to be >10^10^ Jones detectivity) wavelength is thus extended to 2400 nm with this device. In the depletion operation mode, although with much lower responsivity, the device still achieved >10^10^ Jones detectivity up to 2200 nm wavelength and with better noise level control. It is worth mention that such a high detectivity can only be under low illumination level (<1 mW cm^−2^). Considering the light‐dependent responsivity characteristics of the phototransistors (as shown in Figure 4a), one can expect that such devices are most suitable for photodetection applications where the light intensity is weak. With such characteristics, our hybrid phototransistor shows both detectivity and response speed fully compatible to a commercial InGaAs amplified photodetector packaged with a thermoelectric cooler (e.g., PDA10DT as sold by Thorlabs, Inc.) operated in a similar wavelength range but at −10 °C temperature.

In **Table** **1** we also provide a comparison of our hybrid phototransistor with other high‐performance HgTe QD‐based photodetectors reported recently. First, it can be seen that the QD/P3HT combination provides a much faster temporal response, resulting in a great improvement in the response speed of the phototransistor structure. Second, by properly balancing between photoconductive gain and noise current, we achieved a record high detectivity at 2.3 µm. Although currently our QDs can only sense the IR light up to ≈2.5 µm, we believe the material and device strategies can be extended to MWIR (3−5 µm) HgTe QDs and help enhance the room temperature detectivity of the MWIR photodetectors.

**Table 1:** Performance comparison of recently reported HgTe QD photodetectors

	Device structure	Spectral sensing range	Responsivity	D* (with operation temperature)	Decay time (*t*_d_) or 3 dB bandwidth (*f*_3dB_)
This work	P3HT: HgTe QD phototransistor	Up to 2.5 µm	>1 A W^−1^ up to 2250 nm	>10^11^ Jones up to 2300 nm @ RT	*t*_d_ < 1.5 µs *f*_3dB_ > 100 kHz
[4]	HgTe QD phototransistor	Up to 2.3 µm	>0.3 A W^−1^ up to 2200 nm	>10^10^ Jones up to 2200 nm@RT	*t*_d_ < 12.6 µs
[9]	MoS_2_‐HgTe QD phototransistor	Up to 2.1 µm	>100 A W^−1^ up to 2100 nm	≈10^12^ Jones at 2000 nm@RT	*t*_d_ < 4 ms
[14]	HgTe QD photodiode with p‐type HgTe QD layer	Up to 2.5 µm	3 × 10^−4^ A W^−1^	≈3 × 10^8^ Jones@RT	*f*_3dB_ > 10 KHz
[19]	Flexible HgTe QD photodiode with resonant cavity	1.5−2.5 µm	0.2 A W^−1^ @ RT	≈3 × 10^10^ Jones@RT	*t*_d_ = 260 ± 10 ns
[39]	HgTe QD‐ink based photodiode	Up to 2.5 µm	>0.02 A W^−1^@RT	3 × 10^9^ Jones @ RT	*t*_d_ = 370 ns
[17]	Dual band HgTe QD photodiode	1.5−2.5 µm	−	≈5 × 10^10^ Jones@RT	*t*_d_ < 2.5 µs *f*_3dB_ > 100 kHz
		3−5 µm	−	≈10^10^ Jones@150 K	
[16]	HgTe/Ag_2_Te QD photodiode treated with HgCl_2_	4−5 µm	0.56 A W^−1^@160 K	>10^10^ Jones@200 K ≈3 × 10^8^ Jones@RT	*t*_d_ ≈1 µs@85K *t*_d_ ≈50 ns@160 K
[40]	Plasmon resonance enhanced HgTe QD photodiode	3−5 µm	1.6 A W^−1^@85 K	≈10^10^ Jones@220 K ≈7 × 10^8^ Jones@RT	*t*_d_ < 1 µs

In conclusion, we presented a facile method to control the morphology of P3HT: HgTe hybrid phototransistors for efficient infrared photodetection. The HgTe QDs are well dispersed into the P3HT matrix with uniform phase distribution and the combination of materials exhibits efficient charge transport. By shifting the transport path into the conducting polymer and aided by the chemical grafting between the P3HT and the QDs, the response speed, noise level, and sensitivity of the hybrid phototransistor are all significantly improved. A record high of more than 10^11^ Jones detectivity and less than 1.5 µs response time is achieved under room temperature operation, which is fully comparable to the commercial epitaxially grown IR photodetectors operated over a similar wavelength range, but typically at low temperature. Hybrid polymer: HgTe QD high sensitivity and fast response phototransistors demonstrated here, produced using scalable fabrication procedures and with potential compatibility with silicon integrated circuit technology, pave the way toward the commercialization of QD based low‐cost infrared photodetectors.

## Experimental Section

##### Synthesis of HgTe QDs in an Aprotic Solvent

The aprotic solvent, gas‐injection synthesis of HgTe QDs is a modified version of an earlier aqueous synthesis method.^[^ ^1^, ^41^ ^]^ H_2_Te gas was produced by an electrochemical cell (60% concentrated phosphoric acid as electrolyte, platinum wire as anode, and elemental tellurium as cathode). H_2_Te gas buffered in Ar gas was passed over into a solution in the reaction flask. The electrolysis current was supplied by a power supply unit operating in current control mode. The gas outlet from the reaction flask was vented into a measuring cylinder containing a 1 m NaOH solution in order to absorb any excess H_2_Te gas not fully reacted in the reaction flask. Dimethylformamide (DMF) was used as reaction aprotic solvent instead of water in order to minimize the QDs aggregation and so the QDs remain well‐dispersed in the solution. 1.9 g HgCl_2_ was dissolved in 250 mL DMF with 75 mL diphenylether, 5 mL triethylamine, and 2.4 mL furanmethanethiol ligand. The solution mixture was deaerated with Ar gas for 1 h before adding H_2_Te gas to the flow. The reaction was carried out under Ar in a 500 mL three neck reaction flask with a magnetic stirrer. The synthetic process to grow HgTe QDs was separated into nucleation and growth stages. At the nucleation stage, the temperature was controlled at 3 °C with 200 mA electrolysis current for 1 h to synthesize small QDs (with emission between 1200 and 1400 nm). At the growth stage, the temperature was raised gradually to room temperature and then increased to 40 °C. After the temperature became stable, the second growth stage of the synthesis was started with 100 mA electrolysis current and continued until the PL emission reached 2400 nm.

The HgTe QDs taken from the reaction flask were precipitated from the mother solution in an ethyl acetate and hexane mixture for up to 20 min to separate the QDs from any unreacted precursor and then centrifuged to obtain the QD precipitates and the latter then dried in nitrogen gas for 3 min. The precipitates were redissolved in dimethyl sulfoxide (DMSO) under ultrasonication for 2 min. Ligand exchange was then carried out with 5 mL of tetrachloroethylene (TCE), 3 mL dodecanethiol (DDT), and 2 mL formamide. After that, extra DMSO was added to make the volume up to 30 mL. The solution was thoroughly mixed by shaking and then left to stand for 20 min in order to complete the ligand exchange whereby the QDs would separate into a lower TCE rich layer. At this point the HgTe QD solution could be briefly centrifuged to speed up the solution segregation. The lower HgTe QD layer was then taken and precipitated with methanol. The precipitates were dried in nitrogen gas for 10 min and redissolved in a hexane: DDT mixture (200 µL DDT to 100 mL hexane) under ultrasonication for 2 min. After several iterations of precipitation and redissolution, the HgTe QD precipitates were dried a final time in a nitrogen gas stream for 20 min before further use.

##### Preparation of HgTe QD, P3HT: HgTe QD Blend, and P3HT Solutions

For the pristine HgTe QD solution, the HgTe QDs were dissolved after purification in toluene, ultrasonicated for 10 min, and filtered through the 0.45 µm pore filter to remove any aggregated dots before the film deposition. For the P3HT: HgTe QD blends, the P3HT was directly added into the HgTe QD solution described above at a 10 mg mL^−1^ concentration and stirred for at least 4 h at 40 °C. The blends were filtered right before the film deposition. For the pristine P3HT solution, the P3HT was dissolved in toluene at 10 mg mL^−1^, stirred for 4 h at 40 °C, and filtered just before the film deposition.

##### Device Fabrication

The gold interdigitated source–drain electrodes were patterned on the silicon wafers through photolithography, deposited through thermal evaporation, and followed by a lift‐off process. The interdigitated electrodes have a channel length of 5 µm and a total channel width of 15 mm (consisting of 15 repeating unit, each 1000 µm long). The optical sensing area of all the devices reported in this work is 2.45 × 10^−3^ cm^2^. All active layer depositions were performed in a standard nitrogen glovebox. After 15 min of ozone treatment, 50‒60 µL of P3HT: HgTe QDs blend solution was directly dropped onto the prepared silicon wafers, and then spin‐coated and dried at 1000 rpm for 30 s. Next, 240 µL of an EDT: acetonitrile = 1:100 (volume ratio) solution was dropped over the whole QD film and left static for 20 s for ligand exchange to occur and then spin dried at 1000 rpm for 30 s. After that, 240 µL acetonitrile solution was dropped onto the sample and a third spin was started (1000 rpm, 30 s). During the spinning, several drops of acetonitrile solution were dropped on the samples again to rinse out any residual EDT ligands remaining at the surface. This procedure was repeated three times. The as‐deposited films were annealed gently on a 60 °C hot plate for 10 min and then quickly cooled to room temperature. The pristine HgTe QD phototransistor was deposited on the same silicon substrate with a similar multiple spin coating and ligand exchange process as described above and then annealed on a 60 °C hot plate for 10 min. The P3HT transistor was directly spin deposited on the same silicon substrate at 1000 rpm for 30 s and then annealed on a 60 °C hot plate for 10 min.

##### Device Characterization

The PL spectrum of the as‐synthesized HgTe QDs was obtained on an Edinburgh Instruments FLS920P spectrometer system. The IR emission of the QDs was measured with a liquid nitrogen cooled InSb photodiode. The emission was excited by 880 nm light from a steady state diode laser.

The crystal phases of the pristine HgTe QD and P3HT: HgTe QD hybrid layers were identified by an X‐ray diffraction system with a Rigaku ru‐300 diffractometer using Cu*K*_*α*1_ irradiation (*λ* = 1.5406 Å). The FTIR spectra were measured with a Bruker Vertex 70 FTIR spectrometer. The atomic force microscopy (Bruker Dimension Icon) in a tapping mode was employed for the morphology characterization. The SEM images of the film morphologies and the cross‐section of the device structures were made by high‐resolution field emission scanning electron microscopy (ThermoFisher Scientific (FEI), Quanta 400). The TEM images were captured by a field emission transmission electron microscopy (FEI TecnaiF20) at 200 kV.

The current−voltage characteristics were measured with a Keithley 2612 source meter. The light current was recorded with the device illuminated by a Newport LQD1550E 5 mW 1550 nm laser. The light intensity dependent properties were calibrated with the same setup with several near infrared (NIR) absorptive filters. The wavelength‐dependent responsivity was measured with a Keithley 2612 source meter under monochromatic illumination generated by passing the light beam from a 250 W quartz tungsten halogen lamp into a Newport 74125 Oriel Cornerstone 260 1/4 m monochromator. The optical power density was measured to be about 1 mW cm^−2^ at *λ* = 800 nm. The light, passing through a 1500 nm long‐pass filter, was focused by two CaF_2_ lenses onto the samples. A Hamamatsu P5968‐200 InSb photovoltaic detector was used to calibrate the optical power density. The reference InSb photodetector was operated at 80 K with liquid nitrogen cooling.

For transient photocurrent measurements, all devices were biased with a Keithley 2612 source meter and illuminated by a function generator (Agilent 33210A) modulated Newport LQD1550E laser with an 11 Hz repetition frequency. The resulting photocurrent was amplified by a Femto DHPCA‐100 high‐speed current amplifier and recorded with a Tektronix TDS 3014C oscilloscope.

The noise level was measured by an fast Fourier transform (FFT) spectrum analyzer SR760 with silver gel bonded encapsulated devices powered by batteries in a metal electromagnetic shielding box after 10 min stressing under dark conditions in air (power spectral density in dBVrms, Hanning and Blackman‐Harris window, alternating current coupling, average number 1000).

## Conflict of Interest

The authors declare no conflict of interest.

## Supporting information

Supporting Information
