# Supplementary material for: Solution Processed Hybrid Polymer: HgTe Quantum Dot Phototransistor with High Sensitivity and Fast Infrared Response up to 2400 nm at Room Temperature
Source: Adv Sci (Weinh). 2020 May 10;7(12):2000068. doi: 10.1002/advs.202000068 (PMC7312319; doi:10.1002/advs.202000068)
Supplement: Supplementary file 1 — Supporting Information [file ADVS-7-2000068-s001.pdf]

Supporting Information

Solution Processed Hybrid Polymer:HgTe Quantum Dot Phototransistor with High Sensitivity and Fast Infrared Response up to 2400 nm at Room Temperature

Yifan Dong,[†] *Mengyu Chen,*[†] *Wai Kin Yiu, Qiang Zhu, Guodong Zhou, Stephen V. Kershaw, Ning Ke, Ching Ping Wong, Andrey L. Rogach and Ni Zhao**

Dr. Y. Dong
Engineering Research Center of Nano-Geomaterials of Ministry of Education, Faculty of Material Science and Chemistry, China University of Geosciences, Wuhan 430074, China

Dr. Y. Dong, Dr. M. Chen, Dr. Q. Zhu, Dr. G. Zhou, Dr. N. Ke, Prof. C. P. Wong, Prof. N. Zhao
Department of Electronic Engineering, The Chinese University of Hong Kong, Shatin, New Territories, Hong Kong S. A. R.
E-mail: nzhao@cuhk.edu.hk

W. K. Yiu, Dr. S. V. Kershaw, Prof. A. L. Rogach
Department of Materials Science and Engineering and Centre for Functional Photonics (CFP), City University of Hong Kong, Kowloon, Hong Kong S. A. R.

Prof. C. P. Wong
School of Materials Science and Engineering, Georgia Institute of Technology, Atlanta, Georgia, United States.

[†]these authors contributed equally to this work.

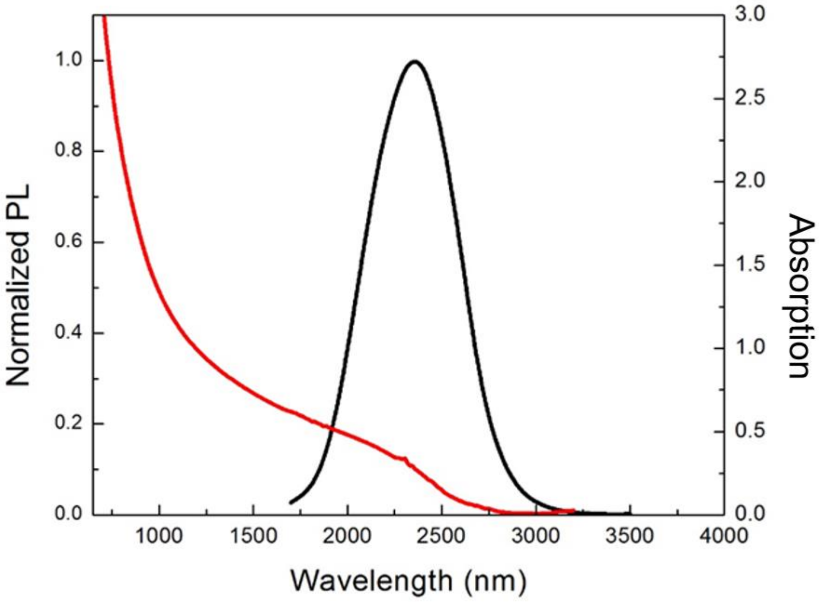

Figure S1. Photoluminescence (PL) and absorption spectra of the as-synthesized HgTe QDs (capped by 1-dodecanethiol and dispersed in tetrachloroethylene).

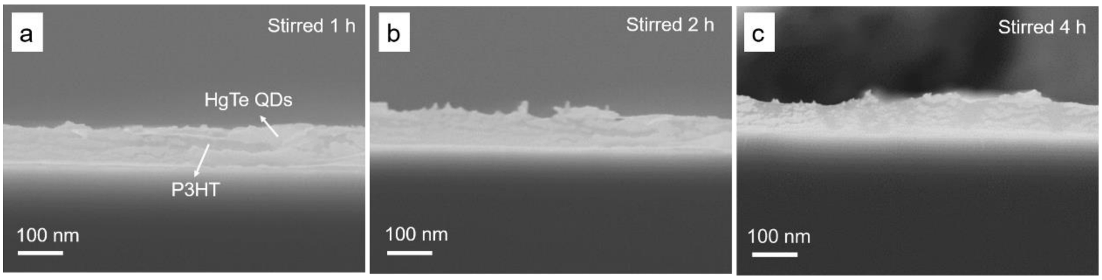

Figure S2. SEM images of the cross-section of three P3HT:HgTe QD film samples with stirring before deposition for (a) one hour, (b) two hours and (c) four hours.

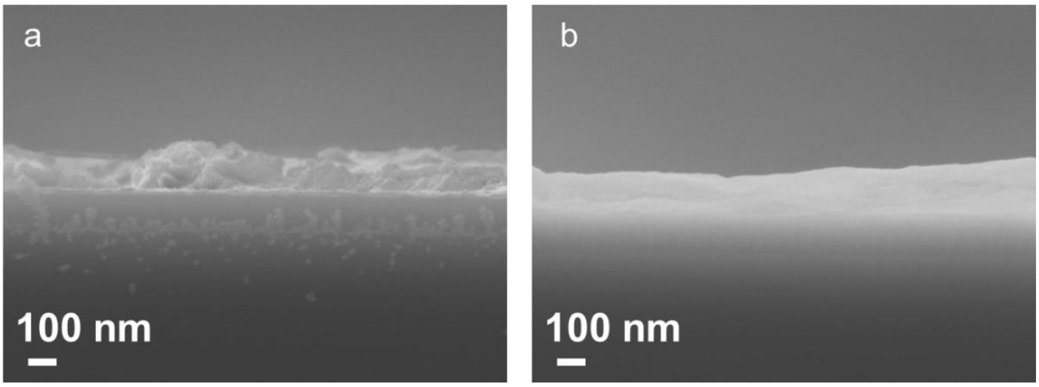

Figure S3. SEM images of the cross-sections of (a) HgTe QD and (b) P3HT transistors.

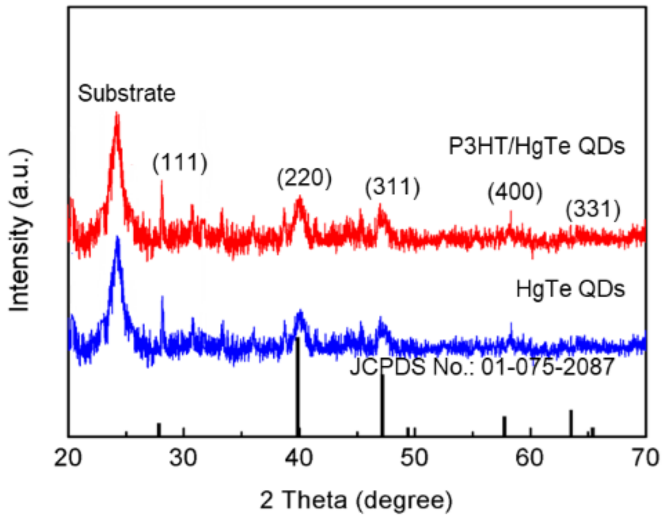

Figure S4. X-ray diffraction (XRD) patterns of HgTe QD and P3HT:HgTe QD hybrid layers. The XRD pattern of the Coloradoite (cubic) phase of HgTe bulk material from the JCPDS database (No. 01-075-2087) is provided for comparison at the bottom.

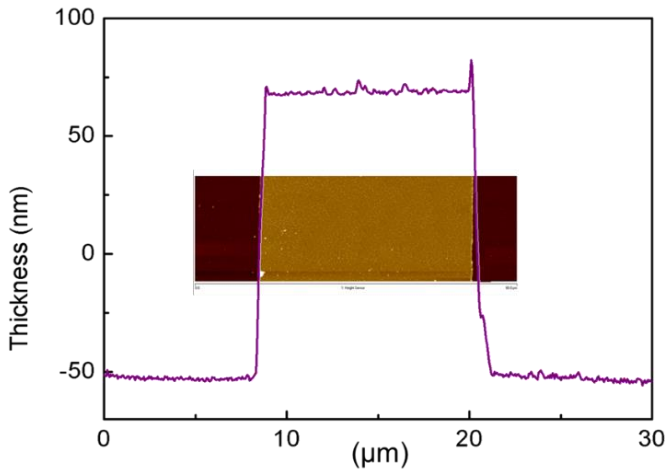

Figure S5. Atomic force microscopy scan of the P3HT:HgTe QD hybrid layer for the thickness and morphology determination.

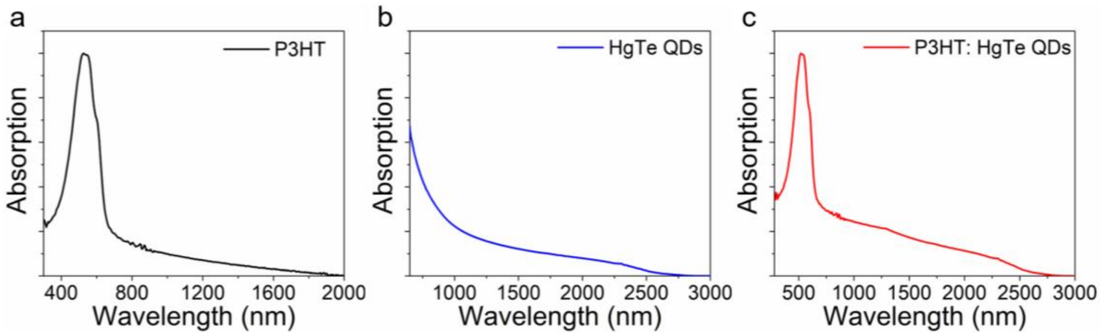

Figure S6. Absorption spectra of the solid films deposited by (a) P3HT, (b) HgTe QDs and (c) P3HT:HgTe QD hybrids.

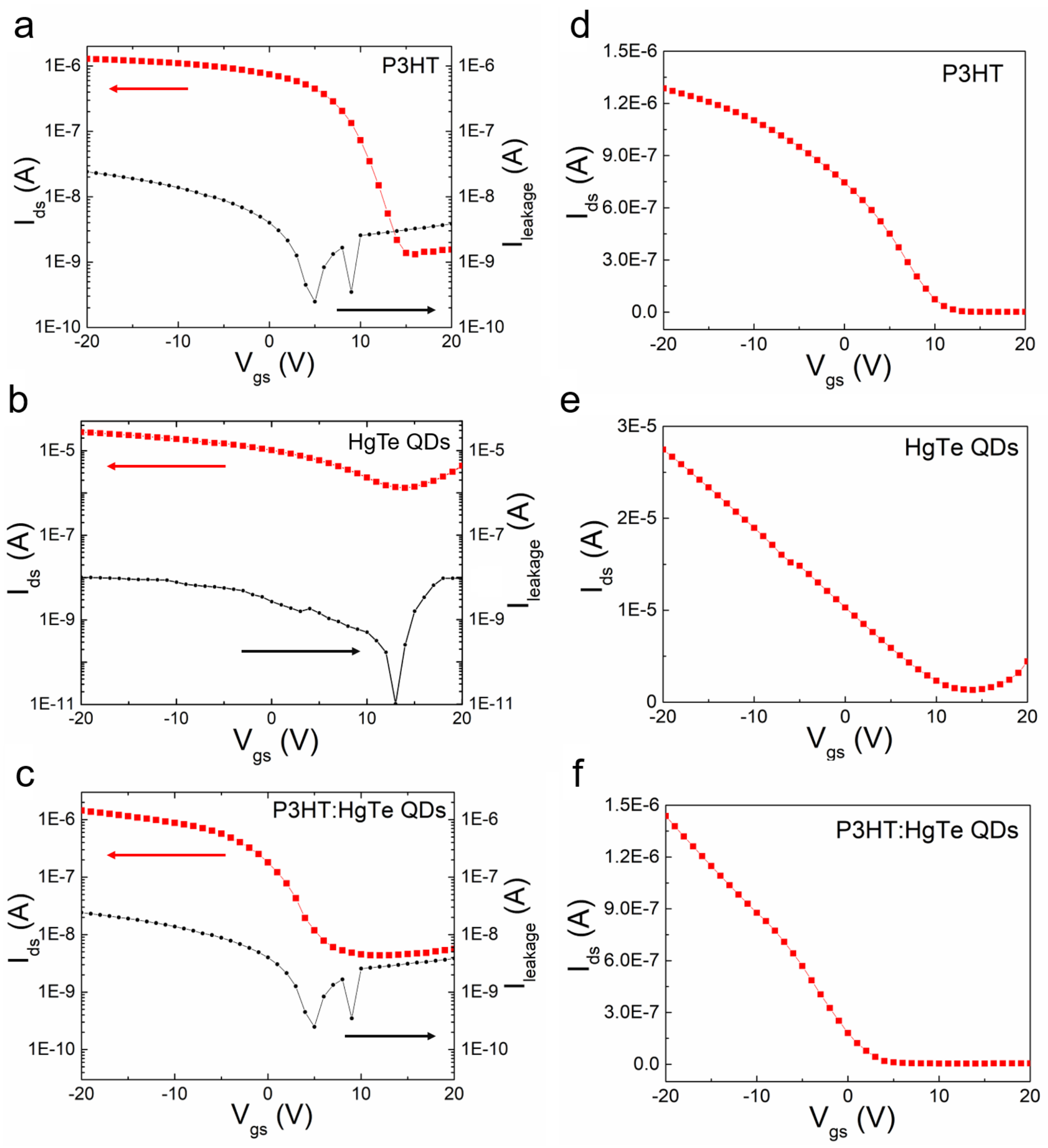

Figure S7. The typical transfer curves and the corresponding leakage currents of (a) P3HT, (b) HgTe QD and (c) P3HT:QD hybrid phototransistors in logarithmic scale. The transfer curves of (d) P3HT, (e) HgTe QD and (f) P3HT:QD hybrid phototransistors are also plotted in linear scale. (all with V_{DS} =

-5V).

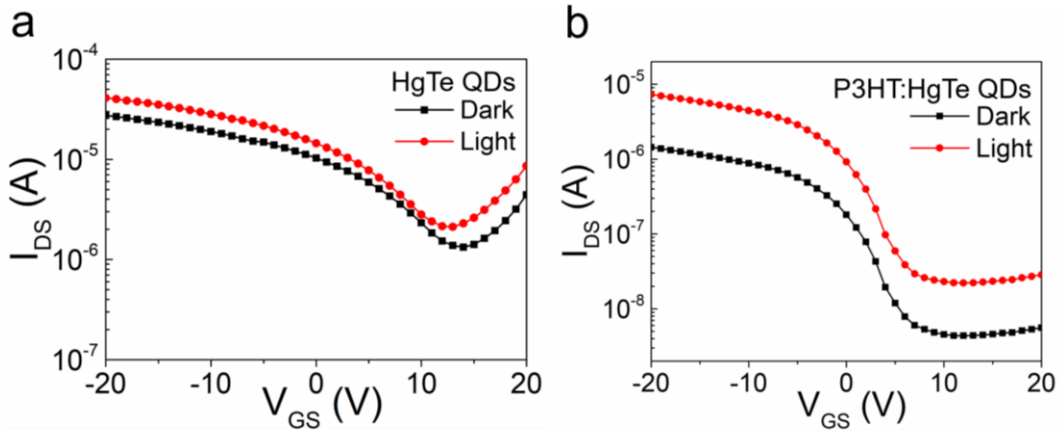

Figure S8. The transfer curves of (a) HgTe QD and (b) P3HT:HgTe QD hybrid phototransistors under dark and illuminated conditions (same devices as in Figure 3 with V_{DS} = -5V, illumination: 1550 nm, 22 mW/cm^2).

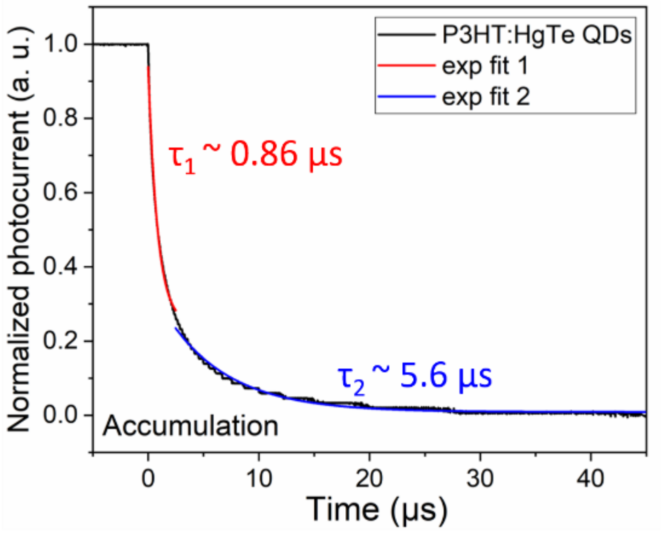

Figure S9. The double exponential fitting of the photocurrent transient decay of P3HT:HgTe QD hybrid phototransistor operated in accumulation mode.

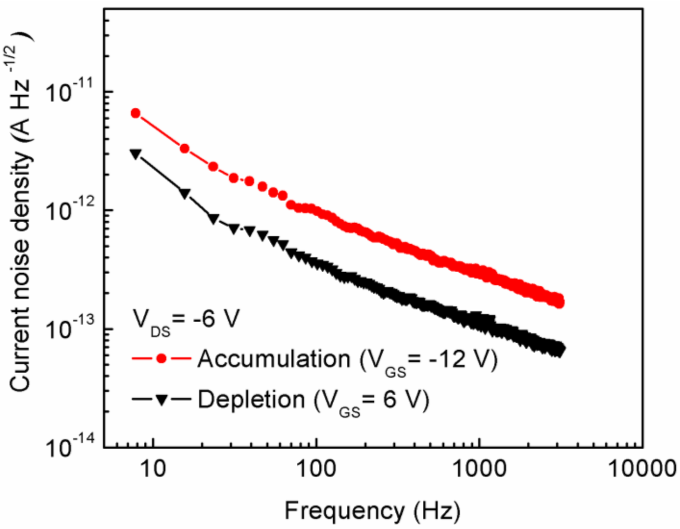

Figure S10. A comparison of the current noise spectral densities of a P3HT:HgTe QD hybrid phototransistor operated in accumulation and depletion modes.
